# The Role of Salicylic Acid in Salinity Stress Mitigation in *Dizygostemon riparius*: A Medicinal Species Native to South America

**DOI:** 10.3390/plants13213111

**Published:** 2024-11-04

**Authors:** Irislene Cutrim Albuquerque, Vitória Karla de Oliveira Silva-Moraes, Givago Lopes Alves, Jordanya Ferreira Pinheiro, Juliane Maciel Henschel, Aldilene da Silva Lima, Priscila Marlys Sá Rivas, Jailma Ribeiro de Andrade, Diego Silva Batista, Fabrício de Oliveira Reis, Tiago Massi Ferraz, Fábio Afonso Mazzei Moura de Assis Figueiredo, Paulo Henrique Aragão Catunda, Thais Roseli Corrêa, Sérgio Heitor Sousa Felipe

**Affiliations:** 1 Programa de Pós–Graduação em Ciências Agrárias, Universidade Estadual do Maranhão, São Luís 65055-310, MA, Brazil; albuquerqueiris0@gmail.com (I.C.A.); vitoriakarlaos@gmail.com (V.K.d.O.S.-M.); engivago@gmail.com (G.L.A.); jordanyaf.p@gmail.com (J.F.P.); priscila.sarivas@gmail.com (P.M.S.R.); jailmarda@gmail.com (J.R.d.A.); diegoesperanca@gmail.com (D.S.B.); fareoli@gmail.com (F.d.O.R.); ferraztm@gmail.com (T.M.F.); figueiredo.uema@gmail.com (F.A.M.M.d.A.F.); thaisroselicorrea@hotmail.com (T.R.C.); 2 Programa de Pós-graduação em Agronomia, Universidade Federal da Paraíba, Areia 58397-000, PB, Brazil; julianemhenschel@gmail.com; 3 Centro de Estudos Superiores de Coelho Neto, Universidade Estadual do Maranhão, Coelho Neto 65620-000, MA, Brazil; aldilene29@gmail.com; 4 Programa de Mestrado Profissional em Rede Nacional em Gestão e Regulação de Recursos Hídricos, Universidade Estadual do Maranhão, São Luís 65055-310, MA, Brazil; paulocatunda.uema@gmail.com; 5 Laboratório de Sementes Florestais, Universidade Estadual do Maranhão, São Luís 65055-310, MA, Brazil

**Keywords:** abiotic stress, bioregulator, *Dizygostemon riparius*, photosynthetic performance

## Abstract

Salicylic acid (SA) is a bioregulator well-known for mitigating salinity damage in plants. However, no studies have examined the interaction between SA and salinity in *Dizygostemon riparius*, a species rich in bioactive molecules. Therefore, we aimed to evaluate the effect of SA application on *Dizygostemon riparius* under different salinity levels. A completely randomized experiment was conducted in a 2 × 3 factorial design (two SA concentrations of 0 and 100 µM and three salinity concentrations of 0, 200, and 400 mM NaCl) with five replicates. At 400 mM NaCl, leaf temperature increased by 11%, while relative water content and total soluble carbohydrates decreased by 30% and 35%, respectively, leading to reduced biomass accumulation. Notably, the SA application mitigated these effects by restoring relative water content under 400 mM NaCl and improving carboxylation efficiency and intrinsic water-use efficiency under 200 mM NaCl. Additionally, dry biomass was maintained under both 200 and 400 mM NaCl with SA treatment. These findings suggest that SA has a promising potential to alleviate salt stress in *Dizygostemon riparius*. Our results could inform cultivation practices, opening new perspectives on the use of SA as an attenuator of salinity stress.

## 1. Introduction

Salinity is a global issue that adversely affects plant growth and development [1,2]. Its impact is multifaceted, as it not only reduces plant productivity but also degrades water quality, diminishes soil biodiversity, and contributes to soil erosion [1]. Currently, it is estimated that the total area of soils affected by salt is 17 million km^2^, occurring mainly in arid and semi-arid regions of Africa, Asia, and Latin America [3]. In Brazil, an estimated 160,000 km^2^ of soil is degraded by salinization and sodification, representing about 2 percent of the nation’s total land area [4]. Specifically in Maranhão state, particularly the Baixada Maranhense region, extensive areas of Solonetz soil are impacted by salinity, limiting agricultural productivity due to high osmotic potential, and soil structure challenges that restrict water availability for plants [5]. Elevated salinity (electric conductivity of the saturated soil solution ≥ 4 dS m^−1^) can affect plants in two distinct phases: the first is the osmotic phase, while the second is the ionic phase [6]. This second phase is the most critical for plant growth and development, causing reduced production or even plant death [6,7].

In the osmotic phase, excess salt in the soil reduces leaf water potential (Ψw), osmotic potential (Ψπ), and turgor pressure (Ψp), leading to osmotic stress, while in the ionic phase, excess salts absorbed by the plant cause a nutritional imbalance and reactive oxygen species (ROS) production (e.g., superoxide anion, O_2_^−^; singlet oxygen, ^1^O_2_; hydroxyl radical, OH^−^; and hydrogen peroxide, H_2_O_2_) [7]. Osmotic stress affects stomatal opening [8], while the accumulation of toxic ions, particularly sodium (Na⁺) and chloride (Cl⁻), later inhibits the absorption of essential nutrients such as potassium (K⁺) and calcium (Ca^2^⁺). These nutrients are crucial for maintaining cellular functions and enzymatic activities involved in photosynthesis [2]. Additionally, ionic toxicity can cause chlorophyll degradation and inhibit the activity of Rubisco (ribulose-1,5-bisphosphate carboxylase/oxygenase), a key enzyme in the photosynthetic process [9].

Among the physiological changes induced by salinity stress is the overproduction of reactive oxygen species (ROS), a group of highly reactive molecules that can damage cellular components, including DNA, proteins, and lipids, leading to lipid peroxidation. [2,10]. To combat this, plants have developed various physiological mechanisms to cope with the stress, both enzymatic (e.g., superoxide dismutase, SOD; catalase, CAT; peroxidase, POX; and glutathione peroxidase, GPX) [11] and non-enzymatic (e.g., ascorbic acid, ASC; reduced glutathione, GSH; and α-tocopherol) systems [12]. In response, the plant accumulates compatible solutes—such as soluble sugars, amino acids, phenolic compounds, and betaines—that enhance cellular osmotic potential, helping the plant retain water and cope with soil salinity [13].

The development of strategies that can mitigate the harmful effects of salinity is one global demand [14]. Based on this, much research has focused on the use of bioregulators, with emphasis on salicylic acid (SA) [15,16,17,18]. SA is a phytohormone synthesized in plants through two primary pathways: phenylalanine ammonia-lyase (PAL) and isochorismate. Its role in plant defense mechanisms under biotic (such as pathogen attack) and abiotic (such as drought, salinity, and extreme temperatures) stress has been widely investigated in several species [19,20,21]. Nonetheless, less is known about SA effects on wild plants rich in bioactive molecules, such as *Dizygostemon riparius* (Plantaginaceae family). In addition to its stress mitigation effect, SA is also known as an elicitor that induces the production of secondary metabolites in plants [22]. Thus, researching the effect of SA on *Dizygostemon riparius* under salinity conditions is the starting point for moving towards an improved cultivation system in line with a better understanding of the impact of salinity on the ecophysiology of this species.

*Dizygostemon riparius* was first cataloged in 2019 in the Cerrado, Maranhão state, Brazil [23]. This species is a subshrub approximately 50 cm in height, ranging from prostrate to erect, with simple to highly branched stems [23,24]. It has oval to elliptical leaves, sparsely pubescent and gland-dotted, with acute to obtuse apexes and crenate-serrate margins [23,24]. Its terminal flowers are subsessile, with pubescent and glandular calyxes and lanceolate sepals [23,24]. Its corollas are white to lilac, with tomentose tubes [23,24]. This species has aroused strong interest in several studies due to its phytochemical potential [25]. The essential oils of this plant species can be used to control the plant disease anthracnose (*Colletotrichum gloeosporioides*) in mango (*Mangifera indica*) fruits [26], to control *Aedes albopictus* larvae [27], and to repel and control the population growth of red spider mites (*Tetranychus neocaledonicus*) [28]. Furthermore, the leaf extract can be used to control *Aedes aegypti* larvae, a vector of arboviruses [29]. In this latest study, phytochemical analysis identified compounds such as polymethoxyflavones and coumarins, with the ethyl acetate extract demonstrating the highest efficacy in larvicidal bioassays (LC_50_ = 542.2 ± 11.5 µg mL^−1^) [29]. Despite its great potential, little is known about its cultivation and management, mainly under adverse conditions, such as saline soils.

Given that less is known about SA effects on wild plants rich in bioactive molecules, such as *Dizygostemon riparius*, researching the effect of SA on this plant under salinity conditions is the starting point for moving towards an improved cultivation system in line with a better understanding of the impact of salinity on the ecophysiology of this species. We aimed to evaluate the effect of two SA concentrations (0 and 100 µM), applied via foliar spray, on *Dizygostemon riparius* under three salinity levels (0, 200, and 400 mM NaCl) and their impact on traits related to ecophysiology and growth.

## 2. Results

### 2.1. Phenotypic Aspects of Plants

**Figure 1:**
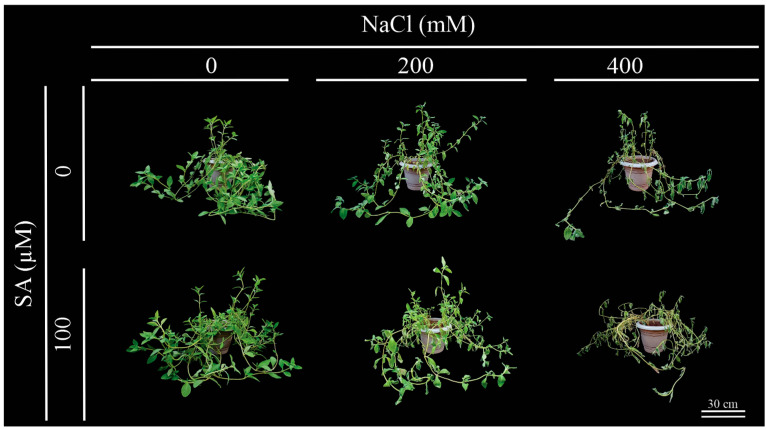
Phenotypic aspects of 66-day-old *Dizygostemon riparius* plants treated without and with salicylic acid (0 and 100 µM for twenty-one days) and grown under different salinity levels (0, 200, and 400 mM NaCl for six days).

*Dizygostemon riparius* plants under salinity (200 and 400 mM NaCl) displayed wilting but few symptoms of leaf chlorosis, in which a slight gradual loss of pigments was evident in older leaves (Figure 1). The wilting response in plants was concentration-dependent and independent of the SA application (Figure 1).

### 2.2. Impact of SA Applications on Plant Ecophysiology During Exposure to Different Salinity Levels

*Dizygostemon riparius* plants without and with the application of SA (0 and 100 µM) and under salinity (200 and 400 mM NaCl, respectively) displayed significant changes in gas exchange parameters from the second day onwards (Figure S1). Plants without SA application and under 200 mM NaCl showed a reduction in net carbon assimilation of 57 and 86% at 4 and 6 days, respectively, while plants with SA application at this same salinity level showed a smaller reduction of 35 and 72% at 4 and 6 days, respectively, when compared to 0 days (Figure S1A). Similarly, plants without SA application and under 400 mM NaCl showed a reduction in net carbon assimilation of 80 and 91% at 4 and 6 days, respectively, while plants with SA application at this same salinity level showed a smaller reduction of 74% at 4 days and there was no mitigation at 6 days in comparison to 0 days (Figure S1A).

Plants without SA application and under 200 mM NaCl showed a reduction in stomatal conductance of 75 and 92% in 4 and 6 days, respectively, while plants with SA application at this same salinity level showed a smaller reduction of 28 and 80% in 4 and 6 days, respectively, when compared to 0 days (Figure S1B). Similarly, plants without SA application and under 400 mM NaCl showed a reduction in stomatal conductance of 83 and 91% in 4 and 6 days, respectively, while plants with SA application at this same salinity level showed a smaller reduction of 73% in 4 days and there was no mitigation in 6 days compared to 0 days (Figure S1B).

Plants without SA application and under 200 mM NaCl showed a reduction in internal CO_2_ concentration of 26 and 27% in 4 and 6 days, respectively, compared to 0 days (Figure S1C). In contrast, plants with SA application at this same salinity level showed a 15% increase in internal CO_2_ concentration at 4 days, compared to 0 days. More strikingly, plants with SA application under 400 mM NaCl showed an increase in internal CO_2_ concentration of 15 and 57% at 4 and 6 days, respectively, compared to day 0 (Figure S1C).

Plants without SA application and under 200 mM NaCl showed a reduction in transpiration rate of 75 and 86% in 4 and 6 days, respectively, while plants with SA application at this same salinity level showed a milder reduction of 31 and 54% in transpiration rate at 4 and 6 days, respectively, compared to 0 days (Figure S1D). At 400 mM NaCl, the plants also followed this same response pattern. Plants without SA application under 400 mM NaCl showed a reduction in transpiration rate of 81 and 81% in transpiration rate at 4 and 6 days, respectively, while plants with SA application at this same salinity level showed a milder reduction of 61 and 78% in transpiration rate at 4 and 6 days, respectively, compared to 0 days (Figure S1D).

Collectively, SA mitigates the deleterious effects of salinity on the gas exchange parameters of plants. In contrast, the maximum quantum yield of photosystem II did not reduce over time, even under high salinity (400 mM NaCl) (Figure S1E).

The statistical results for chlorophyll fluorescence, photosynthetic pigments, gas exchange, dry mass, leaf temperature, and proline and total soluble carbohydrates showed varying levels of significance in 66-day-old *Dizygostemon riparius* plants treated without and with salicylic acid (0 and 100 µM for twenty-one days) and grown under different salinity levels (0, 200, and 400 mM NaCl for six days) on the sixth day of stress (Tables S1–S6).

### 2.3. Impact of SA Applications on Chlorophyll Fluorescence and Photosynthetic Pigments After Six Days of Plant Under Different Salinity Levels

**Figure 2:**
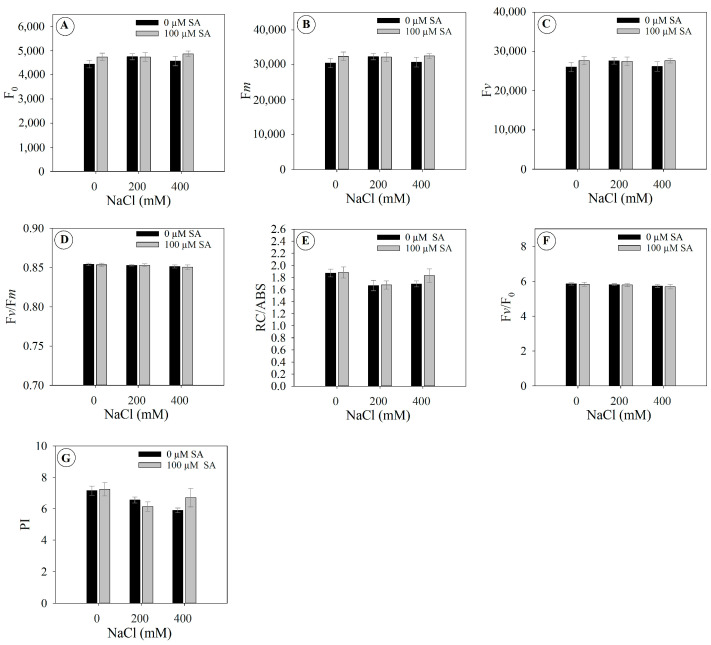
Chlorophyll *a* fluorescence parameter of 66-day-old *Dizygostemon riparius* plants treated without and with salicylic acid (0 and 100 µM for twenty-one days) and grown under different salinity levels (0, 200, and 400 mM NaCl for six days). Values represent means ± standard error (*n* = 5). Means between treatments that are not significantly different are not labeled with letters. (**A**) Initial fluorescence—F_0_; (**B**) maximum fluorescence—F*m*; (**C**) variable fluorescence—F*v*; (**D**) maximum quantum yield of photosystem II—F*v*/F*m*; (**E**) energy absorbed per active reaction center—RC/ABS; (**F**) variable fluorescence per initial fluorescence—F*v*/F_0_, and (**G**) performance index—PI.

The initial fluorescence (F_0_), maximum fluorescence (F*m*) (Figure 2B), variable fluorescence (F*v*) (Figure 2C), maximum quantum yield of photosystem II (F*v*/F*m*) (Figure 2D), energy absorbed per active reaction center (RC/ABS) (Figure 2E), variable fluorescence per initial fluorescence ratio (F*v*/F_0_) (Figure 2F), and the performance index (PI) (Figure 2G) were not significantly modulated, either positively or negatively, in relation to the applied treatments.

**Figure 3:**
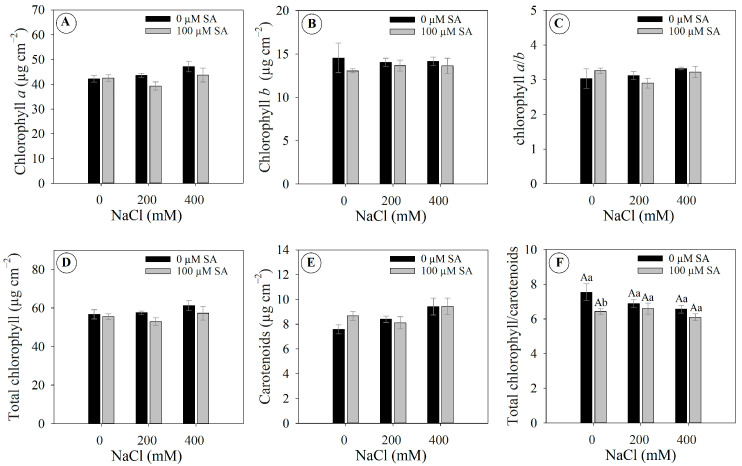
Photosynthetic pigments parameters of 66-day-old *Dizygostemon riparius* plants treated without and with salicylic acid (0 and 100 µM for twenty-one days) and grown under different salinity levels (0, 200, and 400 mM NaCl for six days). Values represent means ± standard error (*n* = 5). Capital letters compare salinity levels within each salicylic acid treatment, while lowercase letters compare the control and salicylic acid treatment within each salinity level (Skoot–Knot test; *p* ≤ 0.05). Means between treatments that are not significantly different are not labeled with letters. (**A**) Chlorophyll *a*; (**B**) Chlorophyll *b*; (**C**) Chlorophyll *a*/*b*; (**D**) Total chlorophyll; (**E**) Carotenoids; and (**F**) Total chlorophyll/carotenoid.

Similarly to the fluorescence response of chlorophyll *a*, the photosynthetic pigments were not significantly affected by the treatments. Chlorophyll *a* value ranged from 39.32 to 47.10 µg cm^−2^ (Figure 3A), chlorophyll *b* ranged from 13.05 to 14.54 µg cm^−2^ (Figure 3B), chlorophyll *a/b* ratio ranged from 2.90 to 3.32 (Figure 3C), total chlorophyll ranged from 52.97 to 61.26 µg cm^−2^ (Figure 3D), and carotenoids ranged from 7.59 to 9.44 µg cm^−2^ (Figure 3E). In contrast, a slight change in the total chlorophyll/carotenoid ratio was observed in plants under 0 mM NaCl, with a significant difference between 0 and 100 µM SA (Figure 3F).

### 2.4. Impact of SA Applications on Gas Exchange After Six Days of Plant Under Different Salinity Levels

**Figure 4:**
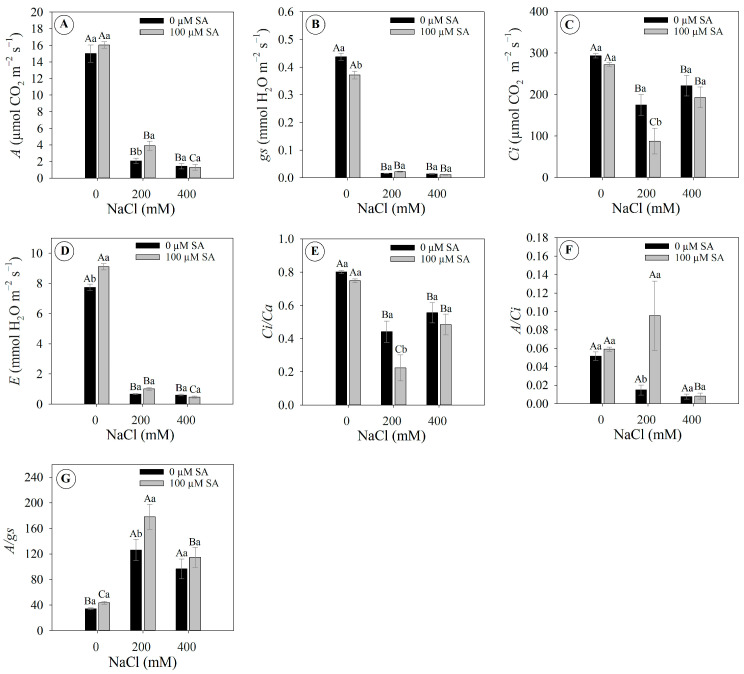
Gas exchange parameters of 66-day-old *Dizygostemon riparius* plants treated without and with salicylic acid (0 and 100 µM for twenty-one days) and grown under different salinity levels (0, 200, and 400 mM NaCl for six days). Values represent means ± standard error (*n* = 5). Capital letters compare salinity levels within each salicylic acid treatment, while lowercase letters compare the control and salicylic acid treatment within each salinity level (Skoot–Knot test; *p* ≤ 0.05). (**A**) Net carbon assimilation—A; (**B**) stomatal conductance—*gs*; (**C**) internal CO_2_ concentration—Ci; (**D**) transpiration rate—*E*; (**E**) ambient intercellular CO_2_ concentration ratio—Ci/Ca; (**F**) carboxylation efficiency—*A*/Ci; and (**G**) intrinsic water-use efficiency—*A*/*gs*.

Regardless of the SA application, 200 and 400 mM NaCl concentrations caused a sharp decrease in the net photosynthetic CO_2_ assimilation, reducing it by 81% and 91%, respectively, compared to the control treatment (or 0 mM NaCl) (Figure 4A). However, specifically at 200 mM NaCl, the application of 100 µM SA in the plants increased net photosynthetic CO_2_ assimilation by 87% compared to the application of 0 µM SA (Figure 4A).

Stomatal conductance to water vapor showed a significant difference only in the treatment with 0 mM NaCl, indicating that foliar application of SA can reduce stomatal conductance in salinity-free environments (Figure 4B). On the other hand, plants under 200 and 400 mM NaCl exhibited a marked reduction of 95 and 97%, respectively, compared to 0 mM NaCl (Figure 4B).

In plants exposed to 200 mM NaCl, the internal CO_2_ concentration decreased with the application of 100 µM SA compared to 0 µM SA (Figure 4C), suggesting that SA application can partially attenuate the deleterious effects of salt stress at this concentration. This finding is further supported by the increase in net photosynthetic CO_2_ assimilation at this salinity level (see Figure 4A).

Plants under 200 and 400 mM NaCl displayed a drastic reduction in transpiration rate of 90 and 94%, respectively, compared to the control (Figure 4D). Interestingly, in plants under 0 mM NaCl, the application of 100 µM SA induced an 18% higher transpiration rate compared to the 0 µM SA treatment (Figure 4D).

Plants under 200 and 400 mM NaCl displayed a reduction in the ambient intercellular CO_2_ concentration ratio of 57 and 33%, respectively, compared to the control (Figure 4E). Specifically for plants under 200 mM NaCl, it was observed that the application of 100 µM SA promoted a 7% reduction in the ambient intercellular CO_2_ concentration ratio compared to the 0 µM SA treatment (Figure 4E).

Plants under 200 mM NaCl salinity displayed a distinct response pattern (e.g., 0 µM SA × 100 µM SA) since the application of 100 µM SA promoted four times more carboxylation efficiency compared to plants without application (or 0 µM AS), indicating a possible mitigation of the deleterious effects on photosynthetic performance (Figure 4F). Similarly, plants exposed to 200 mM NaCl with 100 µM SA application exhibited 41% greater intrinsic water-use efficiency compared to plants without the application (or 0 µM AS) (Figure 4G).

### 2.5. High Salinity Affects Leaf Temperature

**Figure 5:**
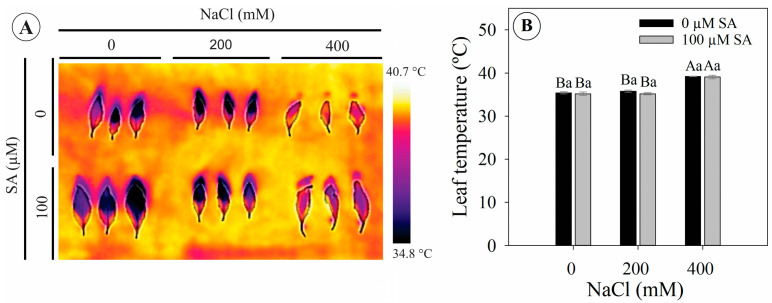
Leaf temperature of 66-day-old *Dizygostemon riparius* plants treated without and with salicylic acid (0 and 100 µM for twenty-one days) and grown under different salinity levels (0, 200, and 400 mM NaCl for six days). Values represent means ± standard error (*n* = 5). Capital letters compare salinity levels within each salicylic acid treatment, while lowercase letters compare the control and salicylic acid treatment within each salinity level (Skoot–Knot test; *p* ≤ 0.05). (**A**) Thermal imaging and (**B**) leaf temperature.

Plants with and without salicylic acid application under 400 mM NaCl exhibited a significant increase of 4 °C in leaf temperature compared to plants under 0 mM NaCl, which corresponds to an increase of 11% (Figure 5A,B).

### 2.6. SA Applications and Salinity Levels Affect Growth and Dry Mass

**Figure 6:**
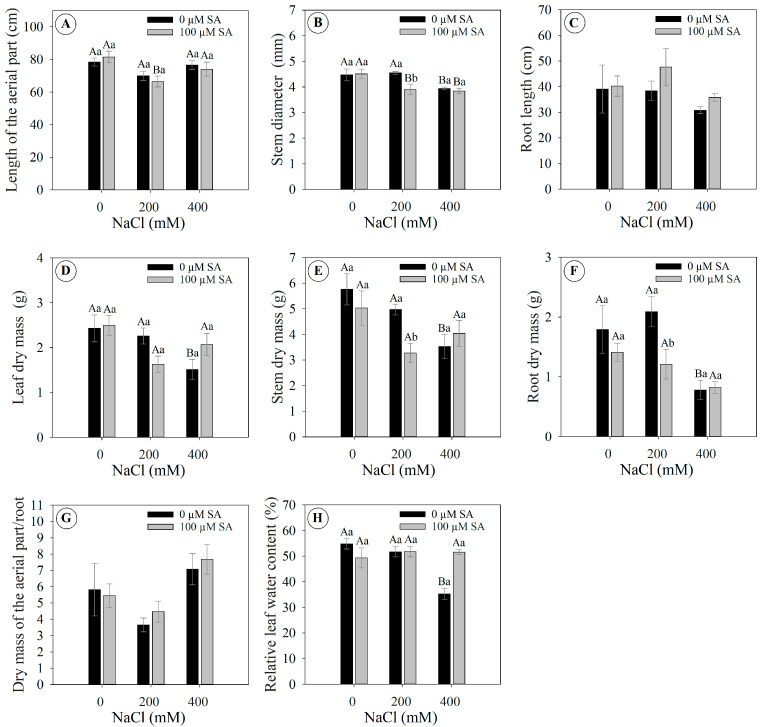
Growth and dry mass parameters of 66-day-old *Dizygostemon riparius* plants treated without and with salicylic acid (0 and 100 µM for twenty-one days) and grown under different salinity levels (0, 200, and 400 mM NaCl for six days). Values represent means ± standard error (*n* = 5). Capital letters compare salinity levels within each salicylic acid treatment, while lowercase letters compare the control and salicylic acid treatment within each salinity level (Skoot–Knot test; *p* ≤ 0.05). Means between treatments that are not significantly different are not labeled with letters. (**A**) Length of the aerial part (cm); (**B**) stem diameter (mm); (**C**) root length (cm); (**D**) leaf dry mass (g); (**E**) stem dry mass (g); (**F**) root dry mass (g); (**G**) ratio between dry mass of the aerial part and root; and (**H**) relative rate of leaf water (%).

The length of the aerial part was not changed in plants without SA application (0 µM AS) at 0, 200, and 400 mM NaCl, while plants with SA application (100 µM SA) displayed a significant reduction of 15% only at 200 mM NaCl compared to the control (0 mM NaCl) (Figure 6A). The stem diameter in plants without SA application (0 µM SA) significantly reduced by 12% only at 400 mM NaCl compared to the control (0 mM NaCl), while plants with SA application (100 µM SA) significantly reduced by 14 and 15% at the concentrations of 200 and 400 mM compared to the control (0 mM NaCl) (Figure 6B). For root length, there was no significant difference between treatments, in which the length varied from 0.78 to 2.09 cm (Figure 6C).

Interestingly, plants without SA application (0 µM SA) significantly reduced leaf dry mass by 62% at 400 mM NaCl compared to the control (0 mM NaCl) (Figure 6D). In contrast, plants with SA application (100 µM SA) did not show significant changes, in which biomass varied from 1.63 to 2.50 g at salinity concentrations (0, 200, and 400 mM NaCl) (Figure 6D). Following this same response pattern, only plants without SA application (0 µM SA) significantly reduced their stem dry mass and root dry mass by 61 and 48%, respectively, while plants with SA application (100 µM SA) did not present significant changes (Figure 6E,F).

The shoot-to-root dry mass ratio tended to show greater biomass accumulation in the shoot compared to the root in plants exposed to 400 mM NaCl, although the differences were not statistically significant (Figure 6G). The relative leaf water content indicated that plants treated with SA (100 µM SA) under 400 mM NaCl were able to maintain water levels similar to the control (0 mM NaCl), while plants without SA application (0 µM SA) in the same conditions displayed a significant reduction of 64% compared to the control (0 mM NaCl) (Figure 6H).

### 2.7. Proline and Total Soluble Carbohydrates Concentrations in Leaves

**Figure 7:**
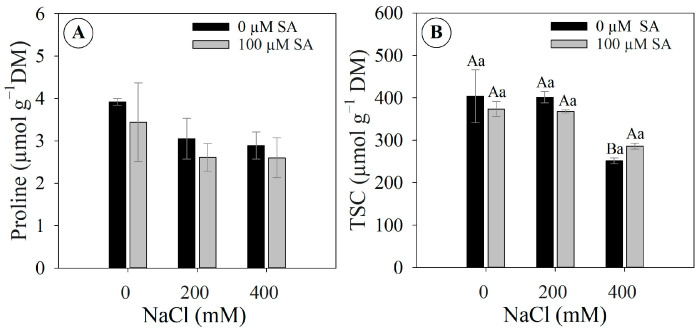
Proline concentration and total soluble carbohydrates (TSC) in leaves of 66-day-old *Dizygostemon riparius* plants treated without and with salicylic acid (0 and 100 µM for twenty-one days) and grown under different salinity levels (0, 200, and 400 mM NaCl for six days). Values represent means ± standard error (*n* = 3). Capital letters compare salinity levels within each salicylic acid treatment, while lowercase letters compare the control and salicylic acid treatment within each salinity level (Skoot–Knot test; *p* ≤ 0.05). Means between treatments that are not significantly different are not labeled with letters. (**A**) Proline and (**B**) total soluble carbohydrates—TSC.

*Dizygostemon riparius* plants with and without SA application under different salinity levels did not exhibit significant changes in proline concentration, which ranged from 1.9 to 5.2 µmol g^−1^ dry mass (Figure 7A), while the concentration of total soluble carbohydrates showed a slight reduction in plants without SA under 400 mM NaCl (Figure 7B).

## 3. Discussion

Saline soil exerts a range of negative effects on plant growth and development, disrupting physiological processes and limiting the ability of plants to achieve optimal biomass and productivity [1,2]. Therefore, this significant global issue necessitates research to evaluate the adaptability strategies of sensitive plants to saline conditions, as well as the potential of salicylic acid (SA) to mitigate the harmful effects of salinity [17,18]. This is the first study to provide ecophysiological insights into *Dizygostemon riparius*, an important source of bioactive molecules, while also assessing its performance under SA application in saline conditions. Our results showed that SA partially mitigated the detrimental effects of salinity, with 100 µM SA maintaining dry mass and relative water content in the leaves of *Dizygostemon riparius* grown under high salinity (400 mM NaCl).

Salinity caused wilting in *Dizygostemon riparius* plants, regardless of SA application. This response is typical under high salinity, as increasing soil salinity leads to a more negative osmotic potential in the soil solution, making water absorption difficult for plants and resulting in a drought-like phenotype [9,30]. Plants exposed to high salinity often wilt due to decreased root hydraulic conductivity, which minimizes the loss of tissue hydraulic integrity [6]. As a result, plant cells lose water, leading to decreased turgor pressure and resulting in a condition known as osmotic stress [31]. Although this phenotypic response was observed, no significant changes were noted in chlorophyll fluorescence or photosynthetic pigments. The ionic toxicity caused by the high influx of Na^+^ and Cl⁻ into the plant can lead to inhibition of Rubisco activity (ribulose-1,5-bisphosphate carboxylase/oxygenase enzymes), decreasing the photosynthetic capacity of plants [9]. Here, gas exchange was strongly reduced from the second day onwards, independently of SA application; however, this was not observed for chlorophyll *a* fluorescence. It is important to highlight that this strong reduction in gas exchange in *Dizygostemon riparius* plants during exposure to salinity is a characteristic of plants classified as glycophytes [32]. Although plants in this group can express biochemical and physiological strategies to deal with salinity stress, they are not always successful [33]. The results indicate that there were no photochemical limitations, as shown by the lack of decrease in fluorescence parameters [34]. Conversely, the strong reduction in carboxylation efficiency (*A*/Ci) and stomatal conductance, followed by decreases in internal CO_2_ concentration and increases in *A*/*gs*, suggest both biochemical and diffusive limitations to photosynthesis [34]. This is in accordance with the results observed for leaf temperature, which is related not only to a drastic reduction in the transpiration rate [31] but also to the accumulation of sodium ions in the leaves, leading to oxidative and thermal stress that causes damage to the photosynthetic apparatus [35,36].

It is noteworthy that the lack of differences in chlorophyll *a* fluorescence and photosynthetic pigment levels were unexpected findings, as a decrease was anticipated in response to salinity. A possible explanation for the maintenance of chlorophyll levels in plants under salinity is the sustained expression of enzymes involved in chlorophyll biosynthesis (such as chlorophyll synthase, Mg-chelatase, and protochlorophyllide oxidoreductase) despite concurrent degradation since these pathways are dynamic in nature [35].

The increase in leaf temperature may be related not only to a drastic reduction in the transpiration rate [31] but also to the accumulation of sodium ions in the leaves, which leads to increased oxidative and thermal stress in the plant in line with possible damage to the thylakoid membranes, increasing heat dissipation [35,36].

The application of salicylic acid (SA) to *Dizygostemon riparius* plants mitigated the harmful effects of salinity on gas exchange, particularly at 200 mM NaCl. Additionally, SA application significantly increased relative water content in plants exposed to 400 mM NaCl and helped maintain dry biomass in plants under both 200 and 400 mM NaCl. Several studies confirm that the application of SA, whether as a pre-treatment or post-treatment, enhances ecophysiological responses against the effects of salinity [37,38,39]. Treatment with SA is also reported to improve plant biomass, chlorophyll content, relative water content, and stomatal conductivity [40], corroborating our findings.

Under high salinity, salicylic acid (SA) triggers a signaling response that induces the accumulation of osmoprotectants (e.g., glycine betaine, proline, and polyamines) and enhances the activities of antioxidant enzymes (e.g., superoxide dismutase [SOD], catalase [CAT], peroxidase [POX], and glutathione peroxidase [GPX]) [38]. This partly explains its use as a powerful bioregulator in mitigating the deleterious effects of salt stress [14]. However, this response is variable; while some plants respond positively by improving their defense against high salinity [37,38,39], others do not appear to be driven to improve their defenses [41,42,43]. Our results did not show a clear modulation of proline levels in plants under salinity; therefore, this amino acid did not serve as either an osmolyte or a protein protector [44] or, possibly, osmoprotection may be mediated by other mechanisms that were not accessed in this study (e.g., glycine betaine and polyamines) [44]. Other studies report that increased proline concentrations may be more related to metabolic disorders induced by severe stress [45].

*Dizygostemon riparius* exhibited salt-sensitive behavior under the salinity levels tested, while salicylic acid (SA) treatment positively affected relative water content and carboxylation efficiency, as well as helped maintain plant dry biomass. This is the first study on the cultivation and management practices of *Dizygostemon riparius*, a recently cataloged species. The current research provides new insights into the ecophysiology of *Dizygostemon riparius*; however, further studies are needed to assess the effects of SA under other salinity levels, as well as at the secondary metabolism level, antioxidant enzyme assays, and indicators such as malondialdehyde, hydrogen peroxide content, and soluble protein, especially to learn if the application of SA can modulate the essential oil profile of this species.

## 4. Materials and Methods

### 4.1. Plant Material and Experimental Conditions

*Dizygostemon riparius* plants were obtained by cloning using the cutting method from mother plants kept in pots in the greenhouse of the Postgraduate Program in Agricultural Sciences of the Maranhão State University, São Luís, Maranhão state, Brazil (2°31′51″ S and 44°18′24″ W). For plant cloning, cuttings measuring 10 cm in length, with two internodes and having four axillary meristems, were selected, in which a bevel cut was made, and the leaves and stem apical meristem were removed. The cuttings were planted in commercial substrate Carolina Soil^®^ (Santa Cruz do Sul, RS, Brazil) and surface forest soil (1:1, *v*/*v*). At 30 days after planting, fertigation was performed with 100 mL in each pot (Plantpar^®^ Indústria e Comércio de Fertilizantes LTDA, Umuarama, PR, Brazil; see Table S7). During the experiment period, the average temperature was ∼29 ± 2 °C, and the relative humidity was 76%, recorded by a digital thermo-hygrometer (Incoterm^®^, Porto Alegre, RS, Brazil) under natural light conditions with an intensity of ~1075 µmol photons m^−2^ s^−1^ and a photoperiod of 12 h.

### 4.2. Salicylic Acid and Salinity Treatments

To test the effect of SA on plants under different salinity conditions, two concentrations (0 and 100 µM SA) were chosen based on previous works with other species rich in bioactive molecules [46]. A total of 15 mL plant^−1^ of both treatments were applied from 45 days of cultivation using a portable plastic sprayer with a capacity of 500 mL (Nobre^®^, Biguaçu, SC, Brazil) until the leaves had wet abaxial and adaxial surfaces. Applications were performed on alternate days, totaling 12 applications for 21 days (until the end of the experiment). The 0 µM AS solution was distilled water only, while the 100 µM SA solution was prepared by dissolving AS in distilled water (Isofar^®^, Duque de Caxias, RJ, Brazil).

**Figure 8:**
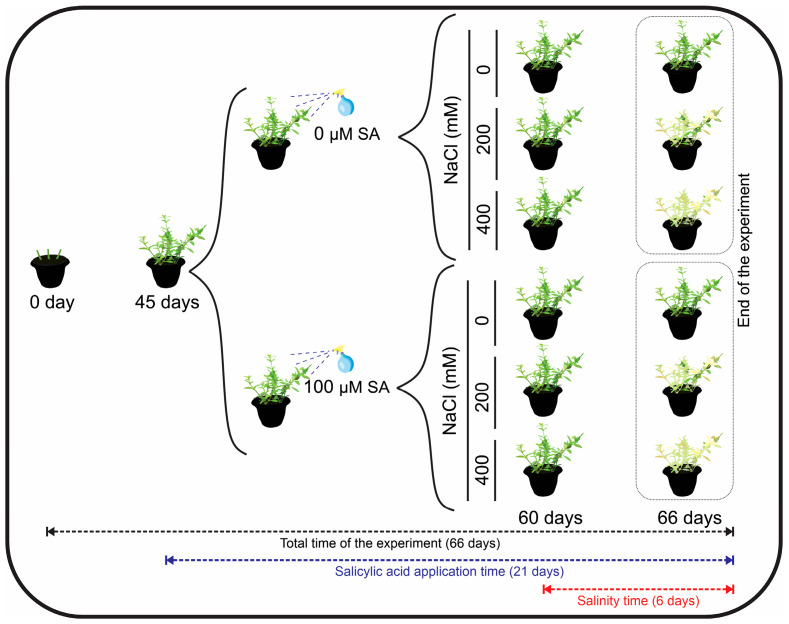
Schematic representation of the experimental design: two salicylic acid concentrations (0 and 100 µM AS) and three salinity concentrations (0, 200 and 400 mM NaCl) on *Dizygostemon riparius*. See further details in Section 4.

At 60 days of plant cultivation, the two groups of plants from the SA treatments (0 and 100 µM AS) were subjected to three concentrations of soil salinity for 6 consecutive days: 0, 200, and 400 mM NaCl (Isofar^®^, Duque de Caxias, RJ, Brazil). These vegetative stages and application times were selected due to the plants’ ability to produce essential oils during this period. NaCl applications occurred gradually until reaching the established concentrations for each treatment (e.g., 100, 200, 300, and 400 mM were applied). The plants were irrigated with saline solution up to 80% of the field capacity. The NaCl solutions prepared with water presented electrical conductivities of 0.06, 13.04, and 18.67 mS cm^−1^ for the concentrations of 0, 200, and 400 mM NaCl, respectively (Gehaka, São Paulo, SP, Brazil) (Figure 8).

### 4.3. Chlorophyll a Fluorescence and Leaf Gas Exchange

Chlorophyll *a* fluorescence and gas exchange measurements were performed on the 4th pair of fully expanded leaves from the shoot apex of the plants at 0, 2, 4, and 6 days in plants under salinity. For fluorescence, leaves were dark adapted for 30 min using specific leaf clips (Hansatech Instrument Ltd., King’s Lynn, Norfolk, UK). This adaptation ensures the complete opening of the reaction centers with minimal heat loss. The parameters of initial fluorescence (F_0_), maximum fluorescence (F*m*), Variable fluorescence (F*v*), maximum quantum yield of photosystem II (F*v*/F*m*), energy absorbed per active reaction center (RC/ABS), Variable fluorescence per initial fluorescence (F*v*/F_0_), and the performance index (PI) were accessed using a portable non-modulated fluorimeter (Pocket PEA, Hansatech Instrument Ltd., King’s Lynn, Norfolk, UK).

Gas exchange analyses were performed using the open gas exchange system Li-6400XT (Li-Cor, Lincoln, NE, USA). The net photosynthetic CO_2_ assimilation (*A*), stomatal conductance to water vapor (*gs*), transpiration rate (*E*), internal CO_2_ concentration (Ci), ambient intercellular CO_2_ concentration ratio (Ci/Ca), carboxylation efficiency (*A*/Ci), and, intrinsic water-use efficiency (*A*/*gs*) were measured between 06:30 and 08:00 h local time under an external CO_2_ concentration of 400 μmol mol^−1^ air and average air temperature of 32 °C. To optimize stomata opening, all measurements were conducted under artificial and saturated light of 1000 μmol m^−2^ s^−1^ produced by a light-emitting diode generating 10% blue light.

### 4.4. Extraction and Quantification of Photosynthetic Pigments

Pigments were extracted from leaf discs 5 mm in diameter, which were obtained from the fourth and fifth pair of leaves fully expanded leaf below the apex. The leaf discs were immersed in 5 mL of dimethyl sulfoxide (DMSO; Êxodo científica^®^, Sumaré, SP, Brazil) and left in the dark for 48 h. Absorbance measurements of the samples at 665 nm, 649 nm, and 480 nm were taken using a UV-visible spectrophotometer (mono-beam) (model UV-M51; BEL Engineering Company, Monza, Italy) in a 10 mm quartz cuvette. The calculation of chlorophyll *a, b*, and carotenoids followed the method outlined by Wellburn [47].

### 4.5. Leaf Thermography

Thermal images of the fourth and fifth pair of leaves from the apex to the base of the plant were captured using a FLIR E8 WIFI thermal imaging camera (FLIR Systems^®^, Wilsonville, OR, USA). The camera was positioned vertically in relation to the leaves at an approximate distance of 100 cm (accuracy of ±2%). Evaluations were carried out between 08:30 and 09:00. The captured images were processed in the FLIR Thermal Studio Suite software version 2.0.x (Copyright^®^, 2024, Thousand Oaks, CA, USA).

### 4.6. Relative Leaf Water Content

Five leaf discs with a 5 mm diameter of fully expanded leaf were collected. The discs were immediately weighed to access the fresh mass (FM), then placed in plastic cups with 8 mL of distilled water for 24 h and then weighed to obtain the turgid mass (TM), then the discs were dried in an oven at 45 °C for 48 h to obtain dry mass (DM). The relative water content was calculated as RLWC (%) = [(FM–DM)/(TM–DM)] × 100 [48].

### 4.7. Growth and Development Analysis

The measurements of the length of the aerial part (cm), stem diameter (mm), and root length (cm) were accessed. Then, the plants were separated by plant organ (leaf, stem, and root) and dried in an oven (SolidSteel^®^, Piracicaba, SP, Brazil) at 45 °C until reaching constant mass. The leaf dry mass (g), stem dry mass (g), root dry mass (g), and the ratio between the dry mass of the aerial part and root were accessed.

### 4.8. Total Carbohydrates Concentration in Leaf

Total soluble carbohydrates (TSC) analysis was performed according to Dubois et al. [49]. An approximately 50 mg dry mass of leaf tissue was macerated and homogenized in 4 mL of distilled water and then vortexed (Ambikontrol^®^, Arujá, SP, Brazil). Subsequently, the material was centrifuged (Eppendorf^®^, Hamburg, Germany) at 3000 rpm for 15 min at 25 °C, and then the supernatant was collected and centrifuged again at 6000 rpm for 10 min. The supernatant was collected and used to quantify TSC. Then, 50 µL aliquots of the extract were removed, and the volume was completed to 0.5 mL with distilled water, adding 50 µL of 5% phenol solution and vortexing, followed by the addition of 2.5 mL of concentrated sulfuric acid. Then, the samples were placed in an ice bath for 10 min to cool and fix the color, the solution was vortexed, and the samples were read using a spectrophotometer (Metash^®^, Shanghai, China) at 490 nm. The CST contents were expressed in µmol carbohydrates g^−1^ of dry mass (DM).

### 4.9. Proline Concentration in Leaf

Proline content was determined following Bates et al. [50] with modifications. An approximately 50 mg dry mass of leaf tissue was macerated and homogenized in 2 mL of 3% sulfosalicylic acid (*w*/*v*), then vortexed and centrifuged at 3500 rpm for 10 min. Then, 0.5 mL of the supernatant was collected in a test tube, added to 0.5 mL of H_2_O, 1 mL of ninhydrin acid (Isofar^®^, Duque de Caxias, RJ, Brazil), and 1 mL of glacial acetic acid (Isofar^®^, Duque de Caxias, RJ, Brazil), and stored at 100 °C for 1 h, stopping the reaction in an ice bath for 10 min. After completion of the reaction, 2 mL of toluene (Isofar^®^, Duque de Caxias, RJ, Brazil) was added, and the mixture was shaken in a vortex test tube shaker. Readings were performed using a spectrophotometer (Metash^®^, Shanghai, China) at a wavelength of 520 nm, and a calibration curve was obtained by preparing standard proline solutions (0–0.150 μmol mL^−1^; Êxodo científica^®^, Sumaré, SP, Brazil) and expressed in µmol g^−1^ dry mass (DM).

### 4.10. Experimental Design and Statistical Analysis

The experiment was conducted in a completely randomized design, in a 2 × 3 factorial scheme [two concentrations of salicylic acid (0 and 100 µM SA) × three salinity levels (0, 200, and 400 mM NaCl)], with five replicates and the experimental unit formed with three plants per pot. The data were tested for normality by the Shapiro–Wilk test, analysis of variance, and the means compared by the Skoot–Knot test (*p* ≤ 0.05) using the Genes software [51].

## 5. Conclusions

Our findings demonstrate that salicylic acid (SA) application at 100 µM effectively mitigates some of the detrimental impacts of salinity on plants *Dizygostemon riparius*, particularly under moderate salinity (200 mM NaCl). SA treatment maintained dry biomass, improved relative water content, and enhanced carboxylation efficiency, suggesting that SA supports the photosynthetic apparatus and water balance under salinity stress. Additionally, the SA application reduced transpiration loss and improved intrinsic water-use efficiency, which is critical for maintaining cellular homeostasis in saline conditions. This study, therefore, highlights the potential of SA as a bioregulator for cultivation in saline environments and provides a foundation for further investigation into its role in supporting the ecophysiology of *Dizygostemon riparius* under adverse conditions. Further studies, particularly at the biochemical level, are necessary to understand how stressful conditions modulate the biosynthesis of secondary metabolites in this species.

## Data Availability

The original contributions presented in the study are included in the article, further inquiries can be directed to the corresponding author.

## Supplementary Materials

The following supporting information can be downloaded at: https://www.mdpi.com/article/10.3390/plants13213111/s1, Figure S1: Gas exchange parameters and maximum quantum yield of photosystem II of *Dizygostemon riparius* plants treated without and with salicylic acid (0 and 100 µM for twenty-one days), and grown under different salinity levels (0, 200, and 400 mM NaCl for six days). Values represent means ± standard error (*n* = 5). A) Net carbon assimilation—A; B) Stomatal conductance—gs; C) Internal CO_2_ concentration—Ci; D) Transpiration rate—E; and E) Maximum quantum yield of photosystem II—Fv/Fm; Table S1: F statistics and levels significance for chlorophyll a fluorescence parameter of 66-day-old *Dizygostemon riparius* plants treated without and with salicylic acid (0 and 100 µM for twenty-one days), and grown under different salinity levels (0, 200, and 400 mM NaCl for six days); Table S2. F statistics and levels significance for photosynthetic pigments parameters of 66-day-old *Dizygostemon riparius* plants treated without and with salicylic acid (0 and 100 µM for twenty-one days), and grown under different salinity levels (0, 200, and 400 mM NaCl for six days); Table S3. F statistics and levels significance for gas exchange parameters of 66-day-old *Dizygostemon riparius* plants treated without and with salicylic acid (0 and 100 µM for twenty-one days), and grown under different salinity levels (0, 200, and 400 mM NaCl for six days); Table S4. Leaf temperature of 66-day-old Dizygostemon riparius plants treated without and with 226 salicylic acid (0 and 100 µM for twenty-one days), and grown under different salinity levels (0, 200, and 400 mM NaCl for six days); Table S5. F statistics and levels significance for growth and dry mass parameters 66-day-old *Dizygostemon riparius* plants treated without and with salicylic acid (0 and 100 µM for twenty-one days), and grown under different salinity 244 levels (0, 200, and 400 mM NaCl for six days); Table S6. F statistics and levels significance for proline concentration and Total soluble carbohydrates (TSC) in leaves of 66-day-old *Dizygostemon riparius* plants treated without and with salicylic acid (0 and 100 µM for twenty-one days), and grown under different salinity levels (0, 200, and 400 mM NaCl for six days); Table S7. Macronutrient and micronutrient composition (%) of fertigation solutions Plantpar Red Flex and Blue Flex. Manufacturer’s recommendation: Add 4.2 g of Blue Flex and 4.2 g of Red Flex per 10 L of water.
