# Supplementary material for: The Role of Salicylic Acid in Salinity Stress Mitigation in Dizygostemon riparius: A Medicinal Species Native to South America
Source: Plants (Basel). 2024 Nov 4;13(21):3111. doi: 10.3390/plants13213111 (PMC11548665; doi:10.3390/plants13213111)
Supplement: Supplementary file 1 [file plants-13-03111-s001.zip › Supplementary Figure.pdf]

Supplementary Information

The role of salicylic acid in salinity stress mitigation in *Dizygostemon riparius*: a medicinal species native from South America

Irislene Cutrim Albuquerque[1]; Vitória Karla de Oliveira Silva–Moraes[1]; Givago Lopes Alves[1]; Jordanya Ferreira Pinheiro[1]; Juliane Maciel Henschel[2]; Aldilene da Silva Lima[3]; Priscila Marlys Sá Rivas[1]; Jailma Ribeiro de Andrade[1]; Diego Silva Batista[1,2]; Fabrício de Oliveira Reis[1]; Tiago Massi Ferraz[1]; Fábio Afonso Mazzei Moura de Assis Figueiredo[1]; Paulo Henrique Aragão Catunda[4,5]; Thais Roseli Corrêa[1]; Sérgio Heitor Sousa Felipe[1*]

[1] Programa de Pós–Graduação em Ciências Agrárias, Universidade Estadual do Maranhão, São Luís 65055–310, Brazil; albuquerqueiris0@gmail.com (I.C.A); vitoriakarlaos@gmail.com (V.K.d.O.S.); engivago@gmail.com (G.L.A.); jordanyaf.p@gmail.com (J.F.P.); priscila.sarivas@gmail.com (P.M.S.R); jailmarda@gmail.com (J.R.d.A.); diegoesperanca@gmail.com (D.S.B.); fareoli@gmail.com (F.d.O.R.); ferraztm@gmail.com (T.M.F.); figueiredo.uema@gmail.com (F.A.M.M.d.A.F.); thaisroselicorrea@hotmail.com (T.R.C.); sergio.h.s.felipe@gmail.com (S.H.F.S.)

[2] Programa de Pós-graduação em Agronomia, Universidade Federal da Paraíba, 58397-000, Areia, PB, Brasil; julianemhenschel@gmail.com (J.M.H.)

[3] Centro de Estudos Superiores de Coelho Neto, Universidade Estadual do Maranhão, Coelho Neto 65620–000, Brazil; aldilene29@gmail.com (A.d.S.L.)

[4] Programa de Mestrado Profissional em Rede Nacional em Gestão e Regulação de Recursos Hídricos, Universidade Estadual do Maranhão, São Luís 65055–310, Brazil; paulocatunda.uema@gmail.com (P.H.A.C.)

[5] Laboratório de Sementes Florestais, Universidade Estadual do Maranhão, São Luís 65055–310, Brazil;

* Correspondence: sergio.h.s.felipe@gmail.com

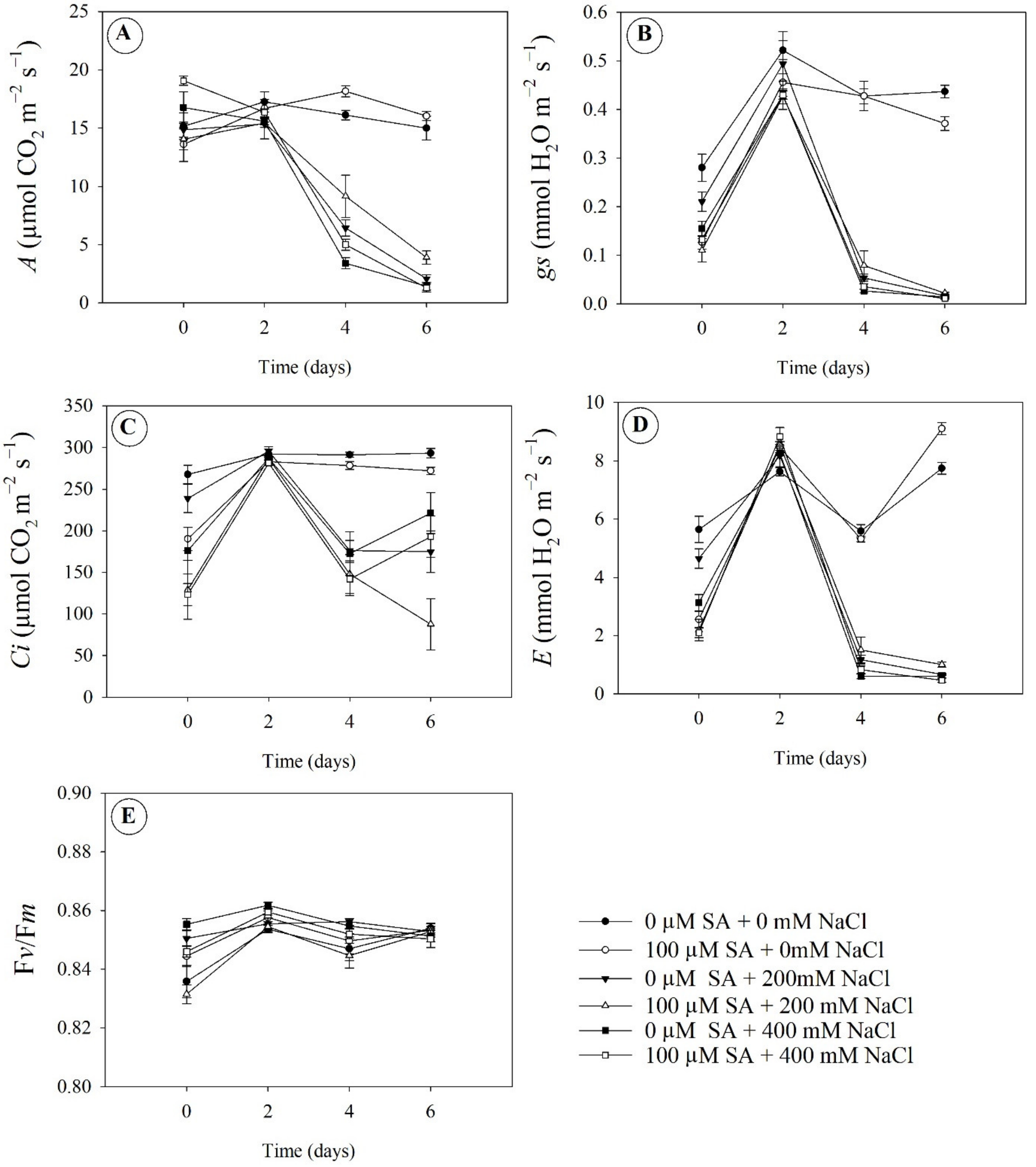

Figure S1. Gas exchange parameters and maximum quantum yield of photosystem II of *Dizygostemon riparius* plants treated without and with salicylic acid (0 and 100 μM for twenty-one days), and grown under different salinity levels (0, 200, and 400 mM NaCl for six days). Values represent means ± standard error (n = 5). A) Net carbon assimilation – A; B) Stomatal conductance – gs; C) Internal CO_2 concentration – Ci; D) Transpiration rate – E; and E) Maximum quantum yield of photosystem II – Fv/Fm.
