# Supplementary material for: The Role of Salicylic Acid in Salinity Stress Mitigation in Dizygostemon riparius: A Medicinal Species Native to South America
Source: Plants (Basel). 2024 Nov 4;13(21):3111. doi: 10.3390/plants13213111 (PMC11548665; doi:10.3390/plants13213111)
Supplement: Supplementary file 1 [file plants-13-03111-s001.zip › Supplementary Tables.pdf]

Supplementary Information

The role of salicylic acid in salinity stress mitigation in *Dizygostemon riparius*: a medicinal species native from South America

Irislene Cutrim Albuquerque[1]; Vitória Karla de Oliveira Silva–Moraes[1]; Givago Lopes Alves[1]; Jordanya Ferreira Pinheiro[1]; Juliane Maciel Henschel[2]; Aldilene da Silva Lima[3]; Priscila Marlys Sá Rivas[1]; Jailma Ribeiro de Andrade[1]; Diego Silva Batista[1,2]; Fabrício de Oliveira Reis[1]; Tiago Massi Ferraz[1]; Fábio Afonso Mazzei Moura de Assis Figueiredo[1]; Paulo Henrique Aragão Catunda[4,5]; Thais Roseli Corrêa[1]; Sérgio Heitor Sousa Felipe[1*]

[1] Programa de Pós–Graduação em Ciências Agrárias, Universidade Estadual do Maranhão, São Luís 65055–310, Brazil; albuquerqueiris0@gmail.com (I.C.A); vitoriakarlaos@gmail.com (V.K.d.O.S.); engivago@gmail.com (G.L.A.); jordanyaf.p@gmail.com (J.F.P.); priscila.sarivas@gmail.com (P.M.S.R); jailmarda@gmail.com (J.R.d.A.); diegoesperanca@gmail.com (D.S.B.); fareoli@gmail.com (F.d.O.R.); ferraztm@gmail.com (T.M.F.); figueiredo.uema@gmail.com (F.A.M.M.d.A.F.); thaisroselicorrea@hotmail.com (T.R.C.); sergio.h.s.felipe@gmail.com (S.H.F.S.)

[2] Programa de Pós-graduação em Agronomia, Universidade Federal da Paraíba, 58397-000, Areia, PB, Brasil; julianemhenschel@gmail.com (J.M.H.)

[3] Centro de Estudos Superiores de Coelho Neto, Universidade Estadual do Maranhão, Coelho Neto 65620–000, Brazil; aldilene29@gmail.com (A.d.S.L.)

[4] Programa de Mestrado Profissional em Rede Nacional em Gestão e Regulação de Recursos Hídricos, Universidade Estadual do Maranhão, São Luís 65055–310, Brazil; paulocatunda.uema@gmail.com (P.H.A.C.)

[5] Laboratório de Sementes Florestais, Universidade Estadual do Maranhão, São Luís 65055–310, Brazil;

* Correspondence: sergio.h.s.felipe@gmail.com

Table S1. F statistics and levels significance for chlorophyll a fluorescence parameter of 66–day–old *Dizygostemon riparius* plants treated without and with salicylic acid (0 and 100 μM for twenty-one days), and grown under different salinity levels (0, 200, and 400 mM NaCl for six days).

Initial fluorescence – F0			
Factors	MS	Fc	p-value
Salinity	4	0.53	0.5912
Salicylic acid	3	2.30	0.1398
Salinity*Salicylic acid	5	0.61	0.54556
Residuals	2		
Total	1		
CV:	8.23 %		
Shapiro-Wilk normality test p-value:	0.3840		

Maximum fluorescence – Fm			
Factors	MS	Fc	p-value
Salinity	3	0.28	0.7607
Salicylic acid	2	1.55	0.2231
Salinity*Salicylic acid	4	0.49	0.6186
Residuals	5		
Total	1		
CV:	8.97 %		
Shapiro-Wilk normality test p-value:	0.6281		

Variable fluorescence – Fv			
Factors	MS	Fc	p-value
Salinity	2	0.27	0.7688
Salicylic acid	5	1.41	0.2450
Salinity*Salicylic acid	3	0.46	0.6374
Residuals	4		
Total	1		
CV:	9.22 %		
Shapiro-Wilk normality test p-value:	0.7377		

Maximum quantum yield of photosystem II – Fv/Fm			
Factors	MS	Fc	p-value
Salinity	3	1.04	0.3657
Salicylic acid	4	0.11	0.7384
Salinity*Salicylic acid	5	0.03	0.9687
Residuals	2		
Total	1		
CV:	0.58 %		
Shapiro-Wilk normality test p-value:	0.5903		

Energy absorbed per active reaction center RC/ABS			
Factors	MS	Fc	p-value

Salinity	5	3.31	0.0499
Salicylic acid	3	0.64	0.4294
Salinity*Salicylic acid	2	0.44	0.6494
Residuals	4		
Total	1		
CV:	11.15 %		
Shapiro-Wilk normality test p-value:	0.3076		

Variable fluorescence per initial fluorescence – Fv/F0			
Factors	MS	Fc	p-value
Salinity	5	1.07	0.3554
Salicylic acid	3	0.10	0.7526
Salinity*Salicylic acid	2	0.01	0.9927
Residuals	4		
Total	1		
CV:	3.89 %		
Shapiro-Wilk normality test p-value:	0.7201		

Performance index – PI			
Factors	MS	Fc	p-value
Salinity	5	4.01	0.0287
Salicylic acid	2	0.30	0.5888
Salinity*Salicylic acid	4	1.54	0.2318
Residuals	3		
Total	1		
CV:	13.11 %		
Shapiro-Wilk normality test p-value:	0.1208		

Table S2. F statistics and levels significance for photosynthetic pigments parameters of 66–day–old *Dizygostemon riparius* plants treated without and with salicylic acid (0 and 100 μM for twenty-one days), and grown under different salinity levels (0, 200, and 400 mM NaCl for six days).

Chlorophyll a			
Factors	MS	Fc	p-value
Salinity	4	2.73	0.0853
Salicylic acid	5	2.83	0.1052
Salinity*Salicylic acid	2	0.95	0.3994
Residuals	3		
Total	1		
CV:	9.21 %		
Shapiro-Wilk normality test p-value:	0.2256		

Chlorophyll b			
Factors	MS	Fc	p-value
Salinity	2	0.01	0.9935
Salicylic acid	5	1.28	0.2698
Salinity*Salicylic acid	3	0.23	0.7950
Residuals	4		
Total	1		
CV:	14.15 %		
Shapiro-Wilk normality test p-value:	0.0033		

Chlorophyll a/b			
Factors	MS	Fc	p-value
Salinity	5	1.47	0.2507
Salicylic acid	2	0.06	0.8110
Salinity*Salicylic acid	4	1.09	0.3531
Residuals	3		
Total	1		
CV:	11.05 %		
Shapiro-Wilk normality test p-value:	0.0103		

Total chlorophyll			
Factors	MS	Fc	p-value
Salinity	3	1.70	0.2041
Salicylic acid	4	3.03	0.0947
Salinity*Salicylic acid	5	0.33	0.7211
Residuals	2		
Total	1		
CV:	8.97 %		
Shapiro-Wilk normality test p-value:	0.4306		

Carotenoids			
Factors	MS	Fc	p-value
Salinity	5	4.27	0.0259
Salicylic acid	2	0.46	0.5051

Salinity*Salicylic acid	4	1.08	0.3559
Residuals	3		
Total	1		
CV:	12.79 %		
Shapiro-Wilk normality test p-value:	0.4465		

Total chlorophyll/carotenoid			
Factors	MS	Fc	p-value
Salinity	4	2.65	0.0910
Salicylic acid	5	6.99	0.0142
Salinity*Salicylic acid	3	1.12	0.3422
Residuals	2		
Total	1		
CV:	9.62 %		
Shapiro-Wilk normality test p-value:	0.0359		

Table S3. F statistics and levels significance for gas exchange parameters of 66–day–old *Dizygostemon riparius* plants treated without and with salicylic acid (0 and 100 μM for twenty-one days), and grown under different salinity levels (0, 200, and 400 mM NaCl for six days).

Net carbon assimilation – *A*			
Factors	MS	Fc	p-value
Salinity	4	0.53	0.5912
Salicylic acid	3	2.30	0.1398
Salinity*Salicylic acid	5	0.62	0.5456
Residuals	2		
Total	1		
CV:	18.79 %		
Shapiro-Wilk normality test p-value:	0.0091		

Stomatal conductance – *gs*			
Factors	MS	Fc	p-value
Salinity	5	1630.48	0.0001
Salicylic acid	3	11.05	0.0028
Salinity*Salicylic acid	4	12.16	0.0002
Residuals	2		
Total	1		
CV:	12.09 %		
Shapiro-Wilk normality test p-value:	0.0010		

Internal CO_2 concentration – *Ci*			
Factors	MS	Fc	p-value
Salinity	5	23.93	0.0001
Salicylic acid	2	6.53	0.0174
Salinity*Salicylic acid	4	1.37	0.2738
Residuals	3		
Total	1		
CV:	23.63 %		
Shapiro-Wilk normality test p-value:	0.3199		

Transpiration rate – *E*			
Factors	MS	Fc	p-value
Salinity	4	2377.64	0.0001
Salicylic acid	5	24.39	0.0001
Salinity*Salicylic acid	3	17.69	0.0001
Residuals	2		
Total	1		
CV:	8.88 %		
Shapiro-Wilk normality test p-value:	0.2935		

Ambient intercellular CO_2 concentration ratio – *Ci/Ca*

Factors	MS	Fc	p-value
Salinity	5	32.72	0.0001
Salicylic acid	4	6.43	0.0181
Salinity*Salicylic acid	3	1.36	0.2748
Residuals	2		
Total	1		
CV:	22.65 %		
Shapiro-Wilk normality test p-value:	0.2796		

Carboxylation efficiency – *A/Ci;*			
Factors	MS	Fc	p-value
Salinity	5	6.01	0.0076
Salicylic acid	4	5.29	0.0304
Salinity*Salicylic acid	3	3.98	0.0323
Residuals	2		
Total	1		
CV:	89.12 %		
Shapiro-Wilk normality test p-value:	0.0001		

Intrinsic water use efficiency – A/gs			
Factors	MS	Fc	p-value
Salinity	3	33.61	0.0001
Salicylic acid	4	5.42	0.0287
Salinity*Salicylic acid	2	1.31	0.2876
Residuals	5		
Total	1		
CV:	31.41 %		
Shapiro-Wilk normality test p-value:	0.2003		

Table S4. Leaf temperature of 66–day–old Dizygostemon riparius plants treated without and with 226 salicylic acid (0 and 100 μM for twenty-one days), and grown under different salinity levels (0, 200, and 400 mM NaCl for six days).

Leaf temperature			
Factors	MS	Fc	p-value
Salinity	5	139.24	0.0001
Salicylic acid	4	2.41	0.1297
Salinity*Salicylic acid	2	0.47	0.6299
Residuals	3		
Total	1		
CV:	1.87%		
Shapiro-Wilk normality test p-value:	0.4961		

Table S5. F statistics and levels significance for growth and dry mass parameters 66–day–old *Dizygostemon riparius* plants treated without and with salicylic acid (0 and 100 µM for twenty-one days), and grown under different salinity 244 levels (0, 200, and 400 mM NaCl for six days)

Length of the aerial part (cm)			
Factors	MS	Fc	p-value
Salinity	3	6.70	0.0049
Salicylic acid	5	0.13	0.7168
Salinity*Salicylic acid	2	0.61	0.5516
Residuals	4		
Total	1		
CV:	9.73%		
Shapiro-Wilk normality test p-value:	0.0604		

Stem diameter (mm)			
Factors	MS	Fc	p-value
Salinity	5	7.44	0.0031
Salicylic acid	4	3.44	0.0758
Salinity*Salicylic acid	3	2.75	0.0838
Residuals	2		
Total	1		
CV:	8.31 %		
Shapiro-Wilk normality test p-value:	0.5578		

Root length (cm)			
Factors	MS	Fc	p-value
Salinity	3	3.07	0.0651
Salicylic acid	2	2.52	0.1257
Salinity*Salicylic acid	4	0.50	0.6125
Residuals	5		
Total	1		
CV:	23.01 %		
Shapiro-Wilk normality test p-value:	0.0190		

Leaf dry mass (g)			
Factors	MS	Fc	p-value
Salinity	4	4.70	0.0189
Salicylic acid	5	0.00	0.9944
Salinity*Salicylic acid	3	3.39	0.0506
Residuals	2		
Total	1		
CV:	24.99 %		
Shapiro-Wilk normality test p-value:	0.2912		

Stem dry mass (g)			
Factors	MS	Fc	p-value
Salinity	5	5.81	0.0087
Salicylic acid	4	2.48	0.1287
Salinity*Salicylic acid	3	2.47	0.1056
Residuals	2		
Total	1		
CV:	25.12 %		
Shapiro-Wilk normality test p-value:	0.2233		

Root dry mass (g)			
Factors	MS	Fc	p-value
Salinity	5	8.04	0.0021
Salicylic acid	4	4.38	0.0472
Salinity*Salicylic acid	3	1.90	0.1710
Residuals	2		
Total	1		
CV:	39.14 %		
Shapiro-Wilk normality test p-value:	0.2001		

dry mass of the aerial part/root			
Factors	MS	Fc	p-value
Salinity	4	6.14	0.0070
Salicylic acid	2	0.20	0.6561
Salinity*Salicylic acid	3	0.21	0.8083
Residuals	5		
Total	1		
CV:	37.25 %		
Shapiro-Wilk normality test p-value:	0.1163		

relative rate of leaf water			
Factors	MS	Fc	p-value
Salinity	3	8.72	0.0014
Salicylic acid	2	3.62	0.0681
Salinity*Salicylic acid	5	11.70	0.0003
Residuals	4		
Total	1		
CV:	10.71 %		
Shapiro-Wilk normality test p-value:	0.2159		

Table S6. F statistics and levels significance for proline concentration and Total soluble carbohydrates (TSC) in leaves of 66–day–old *Dizygostemon riparius* plants treated without and with salicylic acid (0 and 100 μM for twenty-one days), and grown under different salinity levels (0, 200, and 400 mM NaCl for six days).

Total soluble carbohydrates (TSC)			
Factors	MS	Fc	p-value
Salinity	5	11.26	0.0018
Salicylic acid	2	0.09	0.7640
Salinity*Salicylic acid	3	0.97	0.4077
Residuals	4		
Total	1		
CV:	13.23 %		
Shapiro-Wilk normality test p-value:	0.0032		

Proline			
Factors	MS	Fc	p-value
Salinity	4	1.90	0.1919
Salicylic acid	5	2.75	0.1233
Salinity*Salicylic acid	2	0.01	0.9864
Residuals	3		
Total	1		
CV:	25.89 %		
Shapiro-Wilk normality test p-value:	0.6393		

Table S7. Macronutrient and micronutrient composition (%) of fertigation solutions Plantpar Red Flex and Blue Flex. Manufacturer's recommendation: Add 4.2 g of Blue Flex and 4.2 g of Red Flex per 10 liters of water.

Fertigation Plantpar			
Red Fex		Blue Flex	
Nutrients	Quantity (%)	Nutrients	Quantity (%)
N	9.0	N	13.0
Mg	0.6	Ca	15.5
S	3.0	Mg	2.85
P_2O_5	9.0		
K_2O	37.0		
Mn	0.048		
Cu	0.031		
Co	0.002		
Zn	0.019		
B	0.048		
Mo	0.009		
Fe	0.148		
Ni	0.006		
